# Prevalence, patterns, and predictors of prescribing medications for opioid use disorder (MOUD) in the Inpatient setting

**DOI:** 10.1016/j.dadr.2024.100292

**Published:** 2024-11-08

**Authors:** Ashley Burke, Nina Vadiei, Lea Mollon

**Affiliations:** a Banner – University Medical Center South, 2800 Ajo Way, Tucson, AZ 85713, United States; b The University of Texas at Austin, College of Pharmacy, Division of Pharmacotherapy, 8403 Floyd Curl Dr, Austin, TX 78229, United States

**Keywords:** Medications for opioid use disorder, MOUD, Prevalence, Patterns, Predictors, Opioid use disorder, OUD, Inpatient, Hospital

## Abstract

**Background:**

There are many barriers to prescribing medications for opioid use disorder (MOUD). This study evaluates the prevalence, patterns, and predictors of inpatient MOUD prescribing at discharge to patients with a diagnosis of opioid use/opioid use disorder (OUD) that developed opioid withdrawal during their hospital stay.

**Methods:**

This multicenter, retrospective cross-sectional study occurred at three hospitals in Arizona. Patients who developed opioid withdrawal during their hospitalization and had a documented opioid-related disorder between January 1, 2021, and January 1, 2022, were included in the study. Patient-specific factors were evaluated as predictors of MOUD prescribing at hospital discharge using descriptive, multivariate regression.

**Results:**

A total of 382 encounters were included; 249 had documented OUD (65.2 %), 75 of which were discharged with MOUD (75/249; 30.1 %). Patients with moderate/moderately severe opioid withdrawal had higher odds of being discharged with MOUD compared to those with mild opioid withdrawal (OR 2.87 [1.44–5.69], p=0.003). Patients admitted to the largest hospital in Phoenix had higher odds of being prescribed MOUD compared to the largest hospital in Tucson (OR 8.23 [3.02–22.49], p<0.001), as were patients who underwent a routine discharge compared to patient directed discharges (7.63 [2.35–24.71], p=0.001).

**Conclusions:**

Less than one-third of patients with OUD that developed opioid withdrawal during their hospitalization were prescribed MOUD at discharge. Treatment facility, opioid withdrawal severity, and discharge disposition were predictors of MOUD prescribing. Inpatient health-systems and policymakers may consider these data when developing policies/procedures aimed at increasing MOUD prescribing rates.

## Highlights

• Only 30.1 % of patients with OUD that developed opioid withdrawal while hospitalized were discharged on MOUD.
• Treatment facility, opioid withdrawal severity, and discharge disposition were associated with MOUD prescribing.
• Future studies are needed to elucidate what additional factors influence inpatient MOUD prescribing at discharge.

## 1 Introduction

Despite frequent hospitalizations and high healthcare costs (Mark et al., 2013, Ronan and Herzig, 2016, Walley et al., 2012), most inpatients with opioid use disorder (OUD) are not initiated on medications for opioid use disorder (MOUD) during acute encounters (Mauro et al., 2022, Noam et al., 2023, Rosenthal et al., 2023, Thakrar et al., 2021, Christian et al., 2021, Naeger et al., 2016). Patients with substance use disorders, including OUD, often have not engaged in treatment before hospitalization (Englander et al., 2019, Englander et al., 2020), making this a crucial opportunity point for intervention. Unfortunately, treatment teams may overlook this opportunity when patients with OUD are admitted to the hospital for non-opioid related complications and face competing acute care issues.

Numerous barriers to prescribing MOUD exist (Hoffman et al., 2019, National Academies of Sciences, 2019), despite the established efficacy of MOUD in reducing opioid use and overdose risk (Larochelle et al., 2018, Mattick et al., 2014, O'Rourke et al., 2022, Wakeman et al., 2020, Weiner et al., 2024, Liebschutz et al., 2014). These barriers include stigma, inadequate professional education and training, challenges with care coordination, and regulatory or legal obstacles (Jakubowski et al., 2023, National Academies of Sciences, 2019, Reed et al., 2022, Cioe et al., 2020). Additionally, the decision to initiate MOUD in the inpatient setting often relies on a combination of factors, such as provider judgment, past experience, patient preference, and the financial and geographic logistics of available follow-up clinics (Trowbridge et al., 2017). A single-center study by Englander et al. found that other patient-specific factors also predict MOUD initiation in the hospital, such as housing status, whether the patient’s partner also uses substances, whether the patient has previously used methadone, and whether they have concurrent methamphetamine use disorder (Englander et al., 2020). This underscores the importance of investigating additional predictors of MOUD initiation in this setting, as many factors may influence whether a provider chooses to prescribe MOUD, or whether a patient decides to accept treatment.

Previous single-center inpatient studies have reported low rates of MOUD initiation before and after implementation of interventional services that aimed to increase MOUD utilization (Thakrar et al., 2021, Christian et al., 2021). These studies report challenges in identifying hospitalized patients with OUD (Thakrar et al., 2021, Christian et al., 2021), noting the lack of a validated screening tool for OUD that can be administered in acute care settings (Christian et al., 2021). Thakrar et al. found that most patients hospitalized with OUD did not have International Classification of Diseases, 10th revision (ICD-10) codes associated with OUD, indicating that patients with mild opioid withdrawal may be overlooked by treatment teams (Thakrar et al., 2021). Marks et al. similarly noted that a patient’s clinical presentation and prognosis may predict the likelihood of addiction medicine consultation (Marks et al., 2019). This suggests that patients who have never been screened for OUD and who exhibit only mild symptoms of opioid withdrawal during hospitalization may be at greater risk of becoming missed opportunities for intervention. Studies relying on ICD-10 codes to describe MOUD prescribing patterns for patients with OUD likely miss individuals with undiagnosed OUD and milder withdrawal symptoms. Therefore, additional pragmatic studies are needed to more accurately represent hospital-wide MOUD prescribing patterns for all patients experiencing opioid withdrawal during hospitalization, regardless of admission type. Furthermore, multicenter inpatient studies are essential to capture variations in clinical practice between different hospitals.

Thus, the purpose of this multicenter study is to describe MOUD prescribing patterns for hospitalized patients who develop opioid withdrawal and therefore warrant screening/treatment for OUD. Additionally, this study aims to identify predictors of MOUD prescribing at discharge that have not previously been reported, such as the severity of opioid withdrawal during hospitalization, facility type, presence of psychiatric comorbidities, receipt of an addiction medicine consult, discharge disposition type, and patient demographics.

## 2 Materials and methods

### 2.1 Study design

This was a multicenter, retrospective cross-sectional study. Data were collected from three Banner – University Medical Center (BUMC) acute care hospitals across two cities in Arizona (BUMC-Tucson [649-beds]; BUMC-South [245-beds]; BUMC-Phoenix [746-beds]). Patient encounters from January 1, 2021, to January 1, 2022, were included. This time period was intentionally selected since the Coronavirus Disease 2019 (COVID-19) suspended addiction medicine consults at the facilities but resumed in 2021.

### 2.2 Population

Patients were included if they met the following inclusion criteria: 18 years of age or older at the initial encounter; initiated on a Clinical Opiate Withdrawal Scale (COWS) protocol within 24 hours of admission; one or more of the following documented diagnoses with the ICD-10 codes related to opioid use: opioid abuse with intoxication (F11.12), opioid dependence with intoxication (F11.22) or withdrawal (F11.23), opioid use (unspecified) with intoxication (F11.92) or withdrawal (F11.93). Patients with a diagnosis related to opioid use outside of ‘opioid dependence’ (i.e. OUD) were included to minimize the risk of underreporting due to errors in coding (Thakrar et al., 2023). To avoid the same patient being included more than once, only the initial patient encounter was included for patients who had multiple admissions during the study time frame. Patients must have been admitted to the inpatient service rather than the emergency department (ED) to ensure there was opportunity for an addiction medicine consult (hypothesized higher odds of being prescribed MOUD at discharge) (Marks et al., 2019). At the facilities where the study took place, any patient could be started on a COWS protocol by their admitting/primary team to monitor for opioid withdrawal symptoms. Addiction medicine consults were available at all included facilities and could be ordered by the primary treating clinicianat any point during hospital admission regardless of the patient’s location in the hospital. The addiction consult teams (ACTs) are led by an attending physician board-certified in addiction medicine. All three hospitals included in this study are academic medical centers with addiction medicine fellowships; as such, the addiction medicine consult team often comprised of addiction medicine fellows in training. Patients were excluded if they had a chronic pain diagnosis as determined by ICD-10 code G89.4, had an opioid medication documented on their home medication list, or if they had no opioid withdrawal, defined as a COWS score < 5. These exclusion criteria were chosen to avoid including patients where it would have been difficult to determine with certainty whether a prescription for MOUD at discharge would have been for OUD vs. chronic pain (since indications are not always listed on prescriptions). Additionally, patients prescribed opioids prior to admission were excluded so that opioid withdrawal would count as one of the diagnostic criteria for OUD (only two criteria needed for an OUD diagnosis) (Schuckit, 2016, American Psychiatric Association, 2013). Patients that did not have opioid withdrawal were excluded since we aimed to include patients who either had diagnosed OUD or who were more likely to have undiagnosed OUD (i.e., experiencing opioid withdrawal but were not taking prescribed opioids for pain) and were potential candidates for MOUD initiation.

### 2.3 Measures

The electronic health record (EHR) was used to run a system-wide report of patients meeting study inclusion criteria. Duplicate encounters were merged as appropriate, and remaining encounters were validated for meeting study inclusion/exclusion criteria. The following data variables were collected via structured EHR fields: patient age at encounter, sex, race/ethnicity, length of stay, encounter type (inpatient, ED visit), treatment facility, discharge disposition, highest recorded COWS score, hours to first recorded COWS score, ED visits/inpatient admission within 90 days [yes/no], opioid use [yes/no], opioid use disorder [yes/no], psychiatric disorder [yes/no], and MOUD at discharge (methadone, buprenorphine, buprenorphine/naloxone, naltrexone, naltrexone XR). Opioid use and psychiatric disorders were identified via ICD-10 code documentation. Opioid use [yes/no] was measured as patients who had an ICD-10 code for opioid abuse with intoxication (F11.12), or opioid use (unspecified) with intoxication (F11.92) or withdrawal (F11.93); OUD [yes/no] was measured as patients with an ICD-10 code for opioid dependence with intoxication (F11.22) or withdrawal (F11.23). Patients were determined to have a psychiatric disorder if their charted past medical history per ICD-10 codes contained one or more of the following diagnoses: attention deficit hyperactivity disorder (ADHD), adjustment disorder, anxiety, bipolar disorder, borderline personality disorder, brief reactive psychosis, cluster B personality disorder, depression, drug-induced mood disorder, eating disorder, history of attempted suicide/unspecified psychiatric disorder/psychological trauma, hears voices, intermittent explosive disorder, manic-depressive psychosis, mental/mood disorder unspecified, panic attacks, postpartum depression, psychosis, psychotic disorder, post-traumatic stress disorder (PTSD), schizoaffective disorder, schizophrenia, substance-induced psychotic disorder, suicidal behavior/thoughts/attempt.

Additional unstructured data were collected separately via chart review by the first author [AB], specifically pertaining to receipt of an addiction medicine consult [yes/no], which was confirmed by locating a ‘Consult Note’ written by the ACT during the current patient encounter between January 2021 and January 2022. The highest recorded COWS score during the patient encounter was used to categorize the severity of opioid withdrawal (mild: 5–12; moderate: 13–24; moderately severe: 25–36; severe >36). All three hospitals included in this study had long-acting injectable naltrexone available on formulary for OUD treatment.

### 2.4 Data analysis

Statistical analysis was performed using STATA® (V14.2). Categorical data were analyzed using a chi-square test or Fisher’s exact test. Continuous data were analyzed using a Shapiro-Wilk test to determine parametric assumptions and a Student *t* test or Wilcoxon rank sum test to compare parametric means and nonparametric medians, respectively. A multivariable logistic regression was used to identify demographic/clinical factors associated with MOUD prescribing at discharge.

## 3 Results

**Fig. 1:**
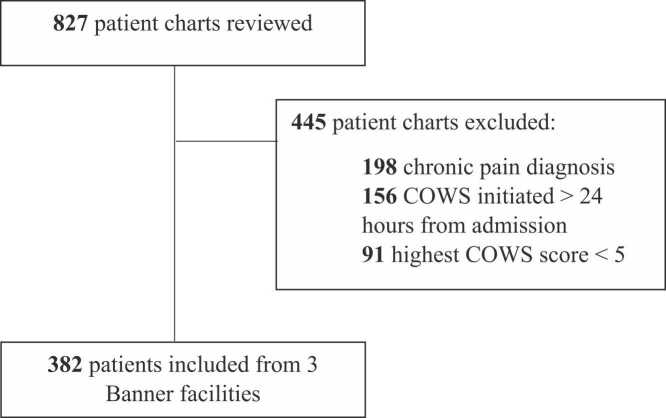
Patient enrollment. COWS: clinical opioid withdrawal scale.

Initial data extraction yielded 3946 patient charts across three Banner facilities. A total of 827 patient records were reviewed, 382 of which met study inclusion criteria (Fig. 1). Of the 382 patients included, 249 had a documented diagnosis of OUD (65.2 %). Of those diagnosed with OUD, 75 were discharged with MOUD (75/249; 30.1 %). Additional demographic and clinical characteristics are reported in Table 1. Most patients were 18–40 years of age (71.5 %), white (85.6 %), and about half of patients were categorized as male (52.4 %). There was a similar proportion of patient encounters included from each hospital, but the majority of prescriptions for MOUD were from BUMC-Phoenix (78.2 %). Most patients who were discharged with MOUD received an addiction medicine consult (65.4 %). About two-thirds of patients had mild opioid withdrawal severity during their admission (68.1 %), and about one-third had moderate withdrawal severity (31.7 %). Most patients had a routine discharge from their initial encounter (62.8 %), while 24.1 % were patient directed discharges. Over one-third of patients had a co-occurring psychiatric diagnosis (38.5 %). Of the 78 MOUD discharge prescriptions, only three prescriptions were for methadone (3.9 %), and none were for naltrexone; the majority were for buprenorphine/naloxone (67.9 %) or buprenorphine (28.2 %).

**Table 1:** Demographic and clinical characteristics.

Characteristic	All patients (n=382)	Discharged with MOUD (n=78)	Discharged without MOUD (n=304)	P-value
Age, years, median (IQR)	28 (28−42)	31 (25−41)	35 (29−42)	0.063
Male, n (%)	200 (52.4)	39 (19.5)	161 (80.5)	0.640
Female, n (%)	182 (47.6)	39 (21.4)	143 (78.6)	
Length of Stay, days, median (IQR)	3 (2−6)	4 (3−8)	3 (2−5)	<0.001
Facility, n (%)				
BUMC-Phoenix	141 (36.9)	61 (43.3)	80 (56.7)	<0.001
BUMC-Tucson	133 (34.8)	9 (6.8)	124 (93.2)	
BUMC-South	108 (28.3)	8 (7.4)	100 (92.6)	
Race/ethnicity, n (%)				
White	327 (85.6)	63 (19.3)	264 (80.7)	0.173
Non-white/other	55 (14.4)	15 (27.3)	40 (72.7)	
Opioid use disorder, n (%)				
No	133 (34.8)	3 (2.3)	130 (97.7)	<0.001
Yes	249 (65.2)	75 (30.1)	174 (69.9)	
Psychiatric Disorder, n (%)				
No	235 (61.5)	43 (18.3)	192 (81.7)	0.194
Yes	147 (38.5)	35 (23.8)	112 (76.2)	
Receipt of addiction medicine consult, n (%)				
No	251 (65.7)	27 (10.8)	224 (89.2)	<0.001
Yes	131 (34.3)	51 (38.9)	80 (61.1)	
Discharge disposition, n (%)				
Routine discharge	240 (62.8)	58 (24.2)	182 (75.8)	<0.001
Patient Directed Discharge	92 (24.1)	4 (4.3)	88 (95.7)	
Other	50 (13.1)	16 (32.0)	34 (68.0)	
Withdrawal Severity (COWS), n (%)				
5–12 (mild)	260 (68.1)	42 (16.2)	218 (83.8)	0.002
13–24 (moderate)	121 (31.7)	35 (28.9)	86 (71.1)	
25–36 (moderately severe)	1 (0.26)	1 (100.0)	0 (0)	
36+ (severe)	0 (0)	0 (0)	0 (0)	
ED Visit at 90 Days, n (%)				
No	291 (76.2)	63 (21.6)	228 (78.4)	0.286
Yes	91 (23.8)	15 (16.5)	76 (83.5)	
IP Admission at 90 Days, n (%)				
No	300 (78.5)	62 (20.7)	238 (79.3)	0.818
Yes	82 (21.5)	16 (19.5)	66 (80.5)	
MOUD Agent at Discharge, n (%)				
Buprenorphine-naloxone	N/A	53 (67.9)	N/A	N/A
Buprenorphine		22 (28.2)		
Methadone		3 (3.9)		
Naltrexone		0 (0)		

IQR: interquartile range; BUMC: Banner University Medical Center; MOUD: medication-assisted treatment; COWS: clinical opioid withdrawal scale; ED: emergency department; IP: inpatient

Multivariable logistic regression (Table 2) revealed higher odds of being prescribed MOUD at discharge if the patient had a documented diagnosis of OUD (OR 5.68 [1.43–22.52], p=0.014) as compared to patients who had an ICD-10 code for opioid abuse or opioid use with intoxication or withdrawal. Patients with moderate/moderately severe opioid withdrawal had higher odds of being prescribed MOUD at discharge compared to those with mild opioid withdrawal (OR 2.87 [1.44–5.69], p=0.003). Patients who went through a routine discharge (7.63 [2.35–24.71], p=0.001) or other non-patient directed discharge (OR 12.88 [2.87–57.82], p=0.001) had higher odds of being prescribed MOUD compared to patient directed discharges. Patients admitted to the largest hospital in Phoenix (BUMC-Phoenix) had higher odds of being prescribed MOUD compared to the largest hospital in Tucson (BUMC-Tucson) (OR 8.23 [3.02–22.49], p<0.001). The odds of receiving MOUD at discharge were numerically higher if there was a documented addiction consult, however, this was not statistically significant (OR 1.96 [0.94–4.06], p=0.072).

**Table 2:** Predictors of MOUD prescribing at hospital discharge.

Variable	OR (95 % CI)	P value
Age	1.01 [0.98–1.04]	0.65
Sex		
Female	Ref	
Male	1.92 [0.96–3.96]	0.077
Race/ethnicity		
White	Ref	
Non-white/other	2.19 [0.92–5.18]	0.088
Facility		
BUMC-Tucson	Ref	
BUMC-South	1.27 [0.41–3.96]	0.68
BUMC-Phoenix	8.23 [3.02–22.49]	<0.001***
Withdrawal Severity		
Mild	Ref	
Moderate-Moderately Severe	2.87 [1.44–5.69]	0.003**
Opioid use disorder		
Yes	5.68 [1.43–22.52]	0.01*
No	Ref	
Psychiatric disorder		
Yes	1.33 [0.70–2.55]	0.39
No	Ref	
Receipt of addiction medicine consult		
Yes	1.96 [0.94–4.06]	0.072
No	Ref	
Discharge disposition		
Patient directed discharge	Ref	
Routine discharge	7.63 [2.35–24.71]	0.001**
Other	12.88 [2.87–57.82]	0.001**
Length of stay	1.02 [0.96–1.10]	0.47

*p<0.05; **p<0.01; ***p<0.001

## 4 Discussion

Studies to date have focused on the impact of MOUD induction in the inpatient setting on reducing the rate of patient directed discharges and/or readmission rates (Noam et al., 2023, O'Rourke et al., 2022, Rosenthal et al., 2023, Wang et al., 2020). To the best of our knowledge, this is the first observational study to describe the patterns and predictors of prescribing MOUD at discharge at multiple hospitals within a large health-system. This study uniquely examined MOUD prescribing patterns for patients diagnosed with opioid use/OUD that experienced opioid withdrawal during their hospitalization, regardless of whether their admitting reason was related to opioid use (e.g., opioid overdose). This population was intentionally included since people with OUD have a higher likelihood of encountering the inpatient system (Walley et al., 2012), and historically most hospitalizations with documented OUD are not secondary to opioid overdose (Raman et al., 2023). Our findings emphasize that there are likely missed opportunities for MOUD prescribing in this population (Raman et al., 2023).

There was a similar distribution of patients admitted to each of the three hospitals included in the study; however, patients admitted to the largest hospital in Phoenix (BUMC-Phoenix) had higher odds of receiving MOUD at discharge compared to the largest hospital in Tucson. There are multiple plausible explanations as to why this may be. According to the Arizona Department of Health Services (ADHS), there is a higher prevalence of patients receiving substance use disorder (SUD) treatment in Phoenix (45 %) than in Tucson (15 %) (Arizona Department of Health Services, 2022). As such, it is possible that coordinating care for prescribing MOUD was more feasible at BUMC-Phoenix. Data was not collected on whether there was an association between prescriber type and MOUD initiation since there were many different prescribers that would have had to be accounted for. Therefore, it’s not possible to ascertain whether the association between treatment facility and MOUD prescribing may be related to differences in prescriber education/training, differences in resource allocation, or other differences that could not be accounted for. Previous studies report multiple barriers to increasing patient access to MOUD both at the individual and health-system level, such as patient/provider stigma, lack of time, increased costs, and regulatory/administrative barriers (e.g., need for prior authorization and low rates of reimbursement) (Jacobson et al., 2020, Mackey et al., 2020, Oliva et al., 2011, Croff et al., 2019, Oliva et al., 2012). Any of these potential barriers could explain the difference in prescribing patterns observed between the hospitals included in this study. Regardless of why these differences exist, these findings suggest an opportunity for health-systems to create or optimize policies/procedures regarding OUD screening and MOUD prescribing. There is an array of publications on implementation strategies for improving access to MOUD (French et al., 2022, Schmidt et al., 2012, Wyse et al., 2022, Croff et al., 2019), but more studies are needed to account for the variability in hospital-specific MOUD prescribing.

Patients with a diagnosis of OUD had higher odds of being prescribed MOUD. This is to be expected since MOUD is indicated for the treatment of OUD. Patients without OUD who had an ICD-10 diagnosis related to opioid use were intentionally included in this study in addition to those with OUD. Thus, this study sample encompasses patients who may have been candidates for MOUD, even if miscoded as having opioid ‘use’ or ‘abuse’ when they really had undiagnosed OUD. This could be considered a study limitation considering MOUD may not be indicated for patients without a documented diagnosis of OUD (34 % of the final study sample). On the other hand, these descriptive data raise the question of what percentage of these 34 % of patients may have had an undiagnosed OUD, particularly if their primary reason for admission was not related to opioid use. This is important to consider since all patient encounters included in this study had a diagnosis related to opioid use, were not prescribed opioids prior to admission, and experienced opioid withdrawal within 24 hours of admission, suggesting a probable likelihood of meeting diagnostic criteria for OUD. Ultimately, these broad inclusion criteria allowed for two key observations: (1) less than one-third of patients diagnosed with OUD in the study sample were prescribed MOUD at discharge, and (2) about one-third of the study sample were diagnosed with ‘opioid use’ or ‘opioid abuse’ rather than OUD, suggesting missed opportunities for OUD screening and treatment.

Patients with moderate/moderately-severe opioid withdrawal severity had higher odds of being prescribed MOUD than patients with mild opioid withdrawal. There are multiple explanations to consider here. It is possible patients with milder symptoms of opioid withdrawal, who are admitted for reasons unrelated to opioid use, are less likely to be screened and treated for OUD. Alternatively, those with diagnosed OUD who are admitted for reasons unrelated to opioid use and present with mild withdrawal symptoms may not have their OUD treatment plan appropriately assessed because it is not the acute treatment priority. Patients with milder withdrawal symptoms may also be less likely to be interested in a prescription for MOUD themselves. However, if prescriber-specific factors play a role in the association between withdrawal severity and MOUD prescribing, establishing protocols to help identify and consult with individuals with OUD in the inpatient setting, regardless of admitting reason or withdrawal severity, may be worth exploring to determine whether such an intervention could help increase MOUD prescribing rates. With adequate staffing support, this type of service could be employed via interprofessional ACTs (Priest and McCarty, 2019). ACTs have been shown to improve care for hospitalized patients with OUD (Martin and Krawczyk, 2024); they increase MOUD initiation, decrease patient directed discharges, and reduce 90-day readmissions (Marks et al., 2019). In the current study, the odds of prescribing MOUD at discharge was numerically higher if the patient received an addiction medicine consult, though these findings were not statistically significant (p=0.072). This may be attributable to differences in ACT operations between the hospitals included in this study. For example, at the time this study took place, the addiction medicine consult service was relatively new at BUMC-South and BUMC-Tucson compared to BUMC-Phoenix. Consequently, the service may have been less known to prescribers of these two hospitals, resulting in underutilization of the service.

Lastly, patients with a routine discharge disposition or other non-patient directed discharge disposition had higher odds of being prescribed MOUD than patient directed discharges. This is understandable considering patient directed discharges pose challenges related to timely care coordination for prescribing MOUD. Regardless, it is noteworthy that about a quarter of patients included in the final study sample had a patient directed discharge, meaning a high volume of patients who may have been candidates for MOUD treatment were potentially missed.

### 4.1 Limitations

There are many limitations of this study that should be taken into consideration. Data classifying the primary admitting reason for each encounter were not collected (e.g., related to opioid use [yes/no]). Since the aim of this study was to broadly describe MOUD prescribing patterns and predictors among all patients admitted to the hospital who developed opioid withdrawal and were diagnosed with opioid use/OUD, there would have been many primary admitting diagnoses to account for, which limited data collection feasibility for this variable. Similarly, data were not collected regarding what hospital unit(s) the patient was assigned to (e.g., intensive care unit, medical/surgical unit, behavioral health unit; all three of which were potential settings in which the current sample may have been located). Future studies investigating whether specific acute care admission types or hospital units are associated with MOUD prescribing would be worth exploring, as it may help identify where more education/intervention is needed. Since our study found significant variability in prescribing patterns between the three hospitals included, it is reasonable to hypothesize that prescribing patterns are associated with differences in prescriber training/education/comfort on MOUD management. However, it should also be noted that data were not available regarding whether MOUD was offered and declined by the patient. Institutions may consider implementing standard operating procedures to document whether MOUD was offered but declined, as this may allow for a better understanding of reasons behind low MOUD prescribing rates.

Data were not collected on whether the patient had received a prescription for MOUD in the past. As such, it is unknown what proportion of the sample prescribed MOUD were new initiations vs. restarting MOUD. Additionally, data were not collected pertaining to whether MOUD was administered in the hospital for the treatment of opioid withdrawal prior to discharge. Future investigators may consider comparing inpatient MOUD use patterns for treating opioid withdrawal vs. continuing MOUD at discharge for ongoing OUD treatment. This could help determine whether there are differences in feasibility between ordering MOUD for short-term treatment of opioid withdrawal vs. prescribing MOUD for ongoing OUD treatment after discharge. It should also be noted that many patients were excluded due to having a chronic pain diagnosis, thus this study does not account for MOUD prescribing patterns and predictors for patients with a chronic pain diagnosis and OUD.

Other limitations of this study are inherent to the retrospective study design, which relies on accurate chart documentation. Fortunately, most of the data collected did not rely on reviewing free text notes. Most patients were categorized as having only mild or moderate opioid withdrawal severity, with only one patient categorized as having moderately-severe opioid withdrawal. Reasons for post-discharge ED visits/inpatient admissions were not investigated. Additionally, ED visits/inpatient admissions could not be identified if people were admitted to a treatment facility outside the ones included in this study. Data were not collected on potential co-occurring SUDs, e.g., stimulant use disorder, alcohol use disorder. Future studies examining predictors of MOUD prescribing may consider collecting these data to determine whether certain co-occurring SUDs predict the likelihood of receiving a prescription for MOUD. Other patient characteristics such as insurance type, income, access to transportation, and housing situation (e.g. homeless [yes/no]) were also unavailable. Lastly, though this study collected data from three facilities at two different cities in Arizona, data are not necessarily generalizable to other regions.

## 5 Conclusion

Less than one-third of patients with OUD that experienced opioid withdrawal during their hospitalization were discharged with a prescription for MOUD. Treatment facility, opioid withdrawal severity, and discharge disposition were predictors of MOUD prescribing. Inpatient health-systems and policymakers may consider these data when developing policies/procedures aimed at increasing MOUD prescribing rates.

## Role of funding source

Nothing declared

## CRediT authorship contribution statement

**Nina Vadiei:** Writing – review & editing, Visualization, Validation, Supervision, Resources, Methodology, Investigation, Formal analysis, Conceptualization. **Lea Mollon:** Writing – review & editing, Validation, Supervision, Resources, Project administration, Methodology, Formal analysis. **Ashley Burke:** Writing – review & editing, Writing – original draft, Visualization, Validation, Methodology, Investigation, Formal analysis, Data curation.

## Declaration of Competing Interest

The authors declare that they have no known competing financial interests or personal relationships that could have appeared to influence the work reported in this paper.
